# Feasibility of National Surveillance of Health-Care-Associated Infections in Home-Care Settings

**DOI:** 10.3201/eid0803.010098

**Published:** 2002-03

**Authors:** Lilia P. Manangan, Michele L. Pearson, Jerome I. Tokars, Elaine Miller, William R. Jarvis

**Affiliations:** Centers for Disease Control and Prevention, Atlanta, Georgia, USA

**Keywords:** home care, surveillance, infection control

## Abstract

This article examines the rationale and strategies for surveillance of health-care-associated infections in home-care settings, the challenges of nonhospital-based surveillance, and the feasibility of developing a national surveillance system.

Over the past 2 decades, the delivery of health care in the United States has shifted increasingly from hospitals to patients’ homes (*1*–*3*). Nearly eight million people in the United States received medical care at home in 1996 (*4*), and an estimated 774,113 (10%) of these patients had at least one indwelling medical device (*5*). Use of a medical device is the greatest predictor (exogenous) of health-care-associated infection.

Home care is often provided by family members who have little or no formal health-care training, which may place patients at increased risk of health-care-associated infections not typically seen in hospitals. In the home-care setting, patients with open wounds or central venous catheters may undertake activities of daily living (e.g., bathing, exercising, gardening, and playing with pets) that may increase the risk of infections.

## Rationale for a National Surveillance System in Home-Care Settings

The epidemiology of health-care-associated infections in home-care settings has not been defined, but infections certainly occur. Outbreaks have been documented in association with use of central venous catheters, parenteral nutrition, bathing practices, educational level of caregivers, and the introduction of new products, such as needleless devices for intravenous infusion (*6*–*8*).

Needleless devices are used for connecting and accessing intravenous infusion tubing, replacing traditional needles. These devices are used in both home and hospital settings and are perceived to be safe for patients and effective in reducing needlestick injuries.

From 1993 through 1995, the Hospital Infections Program, Centers for Disease Control and Prevention (CDC), investigated three outbreaks of bloodstream infections (BSI) in patients receiving infusion therapy in their homes. In all three outbreaks, needleless devices were associated with BSIs. The first outbreak occurred in Rhode Island in 1993-1994. The endcaps on these devices were changed every 7 days. BSIs were frequent when needleless devices were used to administer total parenteral nutrition (*6*). The second outbreak, in Oakland, California, during 1992-1994, occurred among pediatric hematology-oncology patients. The BSI rate was higher when needleless devices were used by Asian or Hispanic children but not by white or black children. The racial/ethnic differences were thought to stem from socioeconomic factors or possibly from language barriers that prevented full understanding of instructions on infection control (*7*). The third outbreak occurred in Houston, Texas, in 1994-1995. The BSI rate was higher when the needleless device endcaps were changed every 7 days and lower when they were changed every 2-3 days. Patients who showered may have had a higher BSI rate than those who took tub baths (*8*).

These outbreak investigations were, by necessity, retrospective, and some data were difficult to obtain. To better define the epidemiology of BSIs in the home-care setting, in 1995 the Hospital Infections Program conducted a prospective multicenter study of home infusion therapy patients. The objectives were to determine rates of BSI and to identify risk factors, especially the use of needleless devices. The study, which was conducted in Cleveland, Ohio, and Toronto, Canada, involved 827 patients (69,532 catheter-days) (*9*). The most common underlying diagnoses among this cohort were infections caused by organisms other than HIV (67%), malignancy (24%), nutritional and digestive disease (17%), heart disease (14%), organ transplantation (11%), and HIV infection (7%).

Overall, 7% of these patients had one or more BSIs during a median of 44 days of catheter use (range 1 to 395 catheter days). A multivariate analysis showed that independent risk factors included recent bone marrow transplant, receipt of total parenteral nutrition, receipt of infusion therapy outside the home (e.g., in a clinic or physician’s office), use of a multilumen catheter, and having had a previous BSI (*9*). Needleless devices were not associated with BSI.

Two prevalence surveys of infections among patients of Missouri home health agencies were conducted by CDC in collaboration with the Missouri Alliance for Home Care (MAHC) and the Missouri Department of Health, the first during summer (June 1-30, 1999) and the second during winter (February 15-March 15, 2000). Of 5,100 home-care patients enrolled in the summer survey, 16% (793) were reported to have infections; 8% (63) of these infections were reported as being acquired at home, 16% (127) as hospital acquired, 35% (278) as unknown source, and 41% (325) as community acquired. The infection sites reported were urinary tract (214 [27%]), respiratory tract (190 [24%]), skin or soft tissue (190 [24%]), surgical site (95 [12%]), or bloodstream (17 [2%]); 18% (143/793) of infections occurred at other body sites (e.g., gastrointestinal, bone) (*10*). Of 2,890 patients enrolled in the winter survey, 16% (466) had infections. The prevalence of respiratory tract infections was higher during the winter survey than during the summer survey. These results suggest that an estimated 1.2 million patients receiving home care in the United States have infections annually, supporting a need for surveillance of infections among home-care patients (*11*).

A nationwide hospital-acquired infection surveillance system and standardized infection definitions have been in existence since the 1970s (*12*–*14*). However, no national surveillance or standardized definitions exist for monitoring infections in the home-care setting. Recently, the Association for Professionals in Infection Control and Epidemiology, Inc. (APIC) published draft definitions for surveillance of infections in home-care patients (*15*). However, these definitions have not yet been validated.

National surveillance of health-care-associated infections in home care may potentially decrease infection rates, as has been documented in hospitals by the National Nosocomial Infections Surveillance system (NNIS). This voluntary, hospital-based reporting system was established to monitor hospital-acquired infections and to guide the prevention efforts of infection control practitioners. During 1990-1999, risk-adjusted infection rates in intensive-care units decreased by approximately 40% among hospitals participating in NNIS (*16*).

A national system for surveillance of health-care-associated infections in home care would not only provide useful data on incidence and types of infections but also simplify identification of risk factors for infection and development of national benchmarks for comparing infection rates. Risk-adjusted rates may assist individual home-care agencies to identify areas for performance and quality improvement and to evaluate the impact of prevention interventions on infection rates.

## Challenges to Developing a National Surveillance System

Home-care surveillance poses several unique challenges, including lack of nationally accepted standard definitions and surveillance methods, loss of patient follow-up, lack of trained infection control personnel in home-care settings, difficulty in capturing clinical and laboratory data, and difficulty in obtaining numerator and denominator data.

### Lack of Nationally Accepted Definitions and Methods

A cornerstone of surveillance in any setting is development of standardized definitions and methods. Individual home-care agencies have developed surveillance definitions for their own use (*17*–*20*), but national definitions of infections in home care do not exist. The draft APIC definitions of home health-care-associated infections have yet to be accepted and implemented nationally. These definitions should be tested to determine their practicality or applicability, given the limited use of laboratory diagnostics in home care. In addition, standard methods of case finding, recording, and calculating rates are also essential. If national benchmark rates are to be established to permit inter- and intra-agency comparisons, consensus definitions of home health-care-associated infections, such as those published by APIC, will have to be implemented.

### Patient Follow-up

Home-care patients often are served by several agencies or are readmitted to the hospital during their illness. Lack of continuity of care hampers detection and reporting of health-care-associated infections. For example, if a home-care patient receiving intravenous therapy has a fever, is admitted to an acute-care facility, and is confirmed to have a BSI, this information may not be communicated to the home-care agency (the same or a different one) when the patient is discharged to continue infusion therapy at home.

### Lack of Trained Personnel

Surveillance requires adequately trained infection control personnel, but few homehealth companies have such employees who are designated to conduct infection control activities, including education, surveillance, and prevention. In a recent survey of home-care agencies in Missouri, only 51 (54%) of 95 had a designated infection control practitioner, and only 27 (53%) of 51 provided ongoing training (*21*). In most home-care agencies, infection control activities are performed on a volunteer basis with no additional compensation. Successful implementation of surveillance programs and other infection control activities in the home health-care setting will require designated and appropriately trained personnel. Training should include calculation of infection rates, recognition of outbreaks and clusters, providing feedback data to essential personnel, and monitoring compliance of prevention efforts. Educational activities targeted at patients, health-care workers, and other caregivers will also be a necessary part of the infection control program.

### Difficulty in Capturing Clinical and Laboratory Data

Many home-care agencies are privately owned and have no hospital or laboratory affiliation; therefore, access to diagnostic services may be limited, and home-care personnel may have difficulties in tracking laboratory results (e.g., contacting out-of-state physician offices or laboratories). Limited access to test results may also encourage home-care personnel to use empiric therapy without documentation of infection or identification of a causative pathogen. Linkages for sharing clinical and laboratory data among physicians, hospitals, and home-care agencies are essential to optimize patient care in the home.

### Difficulty in Obtaining Numerator and Denominator Data

Surveillance for infections in home care will require methods to identify appropriate numerator and denominator data for calculating infection rates for inter- and intra-agency comparison and benchmarks. Collection of numerator data (e.g., BSI or other infectious complications) will require systems that permit data sharing by hospitals and laboratories with home-health agencies.

Capturing appropriate denominator data may even be more difficult (*22*). For example, to determine device-associated infection rates, device utilization must be measured by monitoring days of use. However, if insertion, care, and removal of the device (e.g., central venous catheter, urinary catheter, tracheostomy tube) are done in different health-care settings, it will be difficult to monitor how many days a device is used. Although infection rates based on device utilization have been shown to be necessary in the acute-care setting, it is not certain that they are necessary in home care.

Another option for denominator is the number of days a patient uses a device during home care only, rather than the total number of days (i.e., from insertion to removal) the device is used. Because all home infusion therapy patients have intravenous catheters, patient days may be substituted for device days as long as they equal one another.

In addition to these challenges, the home-care industry will have to deal with the financial implications of implementing and maintaining a national surveillance system. Data on the cost of a surveillance system and on methods of calculating that cost into the reimbursement systems of health-care payors are very much needed.

Despite cost concerns, patient safety and outcomes are becoming increasingly important in the current health-care environment. Purchasers should base their selection of a home-care agency on patient outcomes and satisfaction rather than cost. Thus, home care agencies must conduct surveillance for adverse events. Without such surveillance systems, it would be very difficult for agencies to know if problems are occurring and whether quality care is being provided.

## Progress Toward a National Surveillance System for Health-Care-Associated Infections

Several groups are collecting data on health-care-associated infections in home care and other outpatient areas. These data may prove useful in developing a national home health-care surveillance system.

MAHC is a nonprofit association that provides home care education, advocacy, and information for its 250-member agencies, most of which are located in Missouri. In the early 1990s, MAHC established an infection control committee composed of nurses who provided infection control activities for their agencies. In 1993, the committee implemented the MAHC Infection Surveillance Project (ISP) to monitor infections associated with central venous and urinary catheters. ISP is an active surveillance system that uses standardized criteria and definitions for tracking, aggregating, and reporting urinary infections and BSIs among home-care patients. Currently, 88 home-care agencies from 23 states participate in ISP. Although MAHC has contracted with the Hospital Industry Data Institute, Missouri Hospital Association, to organize and present the ISP data, the results have not yet been published. Although the ISP definitions have not been validated to determine sensitivity and specificity, the data allow participating agencies to compare their infection rates with those of other agencies.

On a broader scale, the Health Care Financing Administration (HCFA), in collaboration with the Center for Health Sciences and Policy Research, has developed the Outcome and Assessment Information Set (OASIS) to measure patient outcomes and improve quality in home care. HCFA requires all Medicare-certified home-care agencies to electronically submit data for their Medicare patients to a central OASIS database in Baltimore, Maryland. The outcomes monitored in OASIS are changes in patient health status, as indicated by need for emergency care or hospitalization, for example. Data collected include patient demographics and medical history, living arrangements, type of wound, urinary tract infection, respiratory devices, medications, emergency care received, transfer to an inpatient facility, and death. Most data items are obtained at start of care, every two calendar months, and at discharge. Since August 1999, eight million records have been entered into the OASIS database, and release of an initial report is anticipated in 2001 after the data are tested for accuracy (HCFA, pers. comm.)

Another national and international data source is the Outpatient Parenteral Antimicrobial Therapy (OPAT) registry, which aims to improve delivery of care and outcomes for outpatients receiving parenteral antimicrobial therapy. OPAT provides a broad database for assessing antimicrobial drug-prescribing practices and outcomes among patients with infections treated in outpatient settings. Data collected include patient demographics, diagnosis, pathogen, venous access device, infusion system, adverse events, clinical outcome, and patient satisfaction. Currently, 23 OPAT provider sites from 19 states are participating in the U.S. registry, and 26 provider sites from 6 countries are in the international registry. OPAT data have been presented at scientific conferences (*23*).

Surveillance methods that are commonly used in hospital programs may not be feasible for home care. Different strategies are needed to make surveillance in the home easier to implement, particularly if adequately trained staff and diagnostic services are limited. For example, the Dialysis Surveillance Network provides a novel way of tracking hospitalization, antimicrobial use, and selected infections in hemodialysis outpatients (*24*). Episodes of potential infection are identified by a clearly defined sequence of steps that involves completing an “incident form” for all patients admitted to a hospital or started on intravenous antimicrobial therapy. The presence (or absence) of symptoms indicating infection is recorded rather than the infections themselves, and a computer algorithm determines whether the infection case definitions are met; the data collector is not required to memorize case definitions. The lessons from this surveillance system, in addition to other traditional outpatient systems, may be useful in establishing national surveillance for home health-care-associated infections.

Nearly as many patients receive home care annually as hospital care. With the continued expansion of home health-care delivery and documented infection risk in this setting, a national system for surveillance of health-care-associated infections in the home-care setting is needed. Collaboration between home health-care agencies, state and federal health agencies, private industry, and national or managed-care organizations is essential to make this system feasible and functional. Development and implementation of such a system would foster better understanding of the epidemiology of health-care-associated infections in the home-care setting. Furthermore, this system would provide a means for monitoring the impact of interventions aimed at preventing the emergence of these infections in the home.
